# Supplementary material for: Climate reverses directionality in the richness–abundance relationship across the World’s main forest biomes
Source: Nat Commun. 2020 Nov 6;11:5635. doi: 10.1038/s41467-020-19460-y (PMC7648646; doi:10.1038/s41467-020-19460-y)
Supplement: Supplementary file 1 — Supplementary Information [file 41467_2020_19460_MOESM1_ESM.pdf]

Supplementary Material

Climate reverses directionality in the richness-abundance relationship across the World's main forest biomes

Jaime Madrigal-González, Joaquín Calatayud, Juan A. Ballesteros-Cánovas, Adrián Escudero, Luis Cayuela, Marta Rueda, Paloma Ruiz-Benito, Asier Herrero, Cristina Aponte, Rodrigo Sagardia, Andrew J. Plumptre, Sylvain Dupire, Carlos I. Espinosa, Olga Tutubalina, Moe Myint, Luciano Pataro, Jerome López-Sáez, Manuel J. Macía, Meinrad Abegg, Miguel A. Zavala, Adolfo Quesada-Román, Mauricio Vega-Araya, Elena Golubeva, Yuliya Timokhina, Markus Stoffel

Supplementary Material S1. Information on regions.

Supplementary Table S1. Geographical, biotic and sampling information for each of the 23 forest regions considered. Values for Longitude, Latitude, Elevation, NPP and Richness at the region level were obtained as the mean of the plot-level values.

Country	Region	Longitude (º)	Latitude (º)	Elevation (m.a.s.l.)	NPP (mean, gDMm^2 yr^{-1})	No. Plots	Plot Size (m^2)	Plot Richness (mean and range)	Plot abundance (mean and range)	Data source
Russia	Kola	39.19	67.58	199.045	551.75	28	400	2.03 (1-4)	105.25 (17-463)	UD
US	Sequoya National Park	-118.41	36.20	2274.69	628.54	132	168.33	2.23 (1-7)	26.21 (1-80)	1
US	Great Canyon National Park	-112.12	36.26	2250.94	658.60	68	168.33	2.54 (1-5)	6.79 (1-22)	1
Sweeden	Northern Sweeden	22.31	66.17	141.40	664.25	101	1256	2.41 (1-6)	55.00 (4-172)	2
Spain	Sierra Nevada National Park	-3.72	38.10	1733.92	831.00	56	1963.49	1.75 (1-4)	148.28 (2-355)	3
Switzerland	Alps	8.89	46.39	1496.74	851.90	234	500.34	2.44 (1-7)	21.85 (1-185)	4
Bhutan	Toepisa	89.78	27.52	2371.11	962.30	160	500.34	4.88 (1-14)	14.90 (1-45)	5
US	Alaska	-133.16	56.30	874.33	980.56	491	168.33	2.17 (1-6)	9.13 (1-30)	1
US	New York	-74.47	43.66	1815.22	988.43	197	168.33	3.29 (1-7)	9.40 (2-24)	1
Brazil	Bahia	-40.90	-9.99	884.37	993.68	106	400	6.09 (2-13)	23.22 (6-77)	6
Ecuador	Western Ecuador	-80.19	-4.22	580.62	1133.59	48	400	12.91 (4-24)	73.41 (13-173)	7
Australia	Victoria	146.80	-37.13	468.11	1146.30	35	900	3.47 (1-7)	50.34 (9-142)	8
France	Mercantour National Park	7.12	44.13	44.13	1302.79	61	706	2.21 (1-5)	33.75 (1-132)	9
Chile	Northern Patagonia	-72.63	-41.59	361.22	1286.75	109	500	3.94 (1-9)	15.67 (1-49)	10
France	Cévennes National Park	3.65	44.26	1123.82	1155.38	98	706	1.88 (1-6)	896.92 (39 3764)	9
Spain	Fuentes Carrionas Natural Park	-4.54	42.93	42.93	1318.21	117	1963.49	1.61 (1-5)	185.92 (1-581)	3
US	Klamath Forest	-123.87	41.78	787.43	1396.67	74	168.33	3.31 (1-7)	37.87 (2-90)	1
Ecuador	Podocarpus National Park	-79.01	-4.12	4.12	1549.45	30	1000	73.33 (32-121)	355.76 (180-653)	11
Peru	Río Abiseo National Park	-77.38	-7.64	1948.80	1659.63	30	1000	68.93 (26-125)	382.26 (192-645)	11
Myanmar	Wetphuyay	96.54	20.72	1095.66	1669.19	62	500.34	4.74 (1-18)	12.43 (1-87)	12
Uganda	National Park	31.54	1.76	1042.00	1799.10	622	100	4.69 (1-13)	7.30 (1-19)	13
Bolivia	Madidi National Park	-67.94	-14.21	656.25	2095.54	44	1000	71.81 (48-109)	310.31 (193-522)	14
Costa Rica	Costa Rica	-84.15	9.87	933.97	2274.26	44	1000	20.28 (1-41)	43.26 (2-86)	15

UD unpublished data. See Supplementary material S7 for further details.

Supplementary Table S2. Standardized estimates for every supported Structural Equation Models at the regional level. DF: degrees of freedom; Std. Estimates: Standardized regression coefficients.

Country	Region	Response	Predictor	DF	Std. Estimates
US	Alaska	abundance	richness	346	0.5551
		abundance	mean DBH	346	-0.2105
		abundance	elevation	346	0.0598
		richness	elevation	348	-0.2185
		mean DBH	elevation	348	-0.198
France	Cévennes	abundance	mean DBH	95	-0.5206
		abundance	elevation	95	-0.0402
		richness	abundance	95	-0.0036
		richness	elevation	95	0.0406
		mean DBH	elevation	96	-0.0952
France	Mercantour	abundance	mean DBH	58	-0.5506
		abundance	elevation	58	-0.1509
		richness	abundance	58	0.2979
		richness	elevation	58	-0.5574
		mean DBH	elevation	59	0.1161
Switzerland	Alps	abundance	richness	230	-0.0168
		abundance	mean DBH	230	-0.5199
		abundance	elevation	230	-0.027
		richness	elevation	232	-0.1199
		mean DBH	elevation	232	0.1198
Spain	Fuentes Carrionas Natural Park	abundance	mean DBH	114	-0.6747
		abundance	elevation	114	-0.1245
		richness	abundance	114	0.0597
		richness	elevation	114	-0.1314
		mean DBH	elevation	115	0.0056
Spain	Sierra Nevada National Park	abundance	mean DBH	53	-0.5466
		abundance	elevation	53	0.1214
		richness	abundance	53	0.4474
		richness	elevation	53	-0.0369
		mean DBH	elevation	54	0.1178
USA	New York	abundance	richness	145	0.3182
		abundance	mean DBH	145	-0.2721
		abundance	elevation	48	0.1875
		richness	elevation	48	0.0564
		mean DBH	elevation	48	-0.2309
US	Great Canyon National Park	abundance	richness	162	0.5456
		abundance	mean DBH	162	-0.1017
		abundance	elevation	63	0.0877
		richness	elevation	63	0.2013
		mean DBH	elevation	63	-0.0004

Country	Region	Response	Predictor	DF	Std. Estimates
US	Sequoya National Park	abundance	mean DBH	129	-0.0403
		abundance	elevation	129	0.0758
		richness	abundance	129	0.4675
		richness	elevation	129	-0.2226
		mean DBH	elevation	130	0.5335
US	Klamath National Forest	abundance	richness	70	0.6619
		abundance	mean DBH	70	0.0555
		abundance	elevation	70	-0.0658
		richness	elevation	72	0.0295
		mean DBH	elevation	72	0.0366
Australia	Victoria	abundance	richness	40	0.1628
		abundance	mean DBH	40	-0.6058
		abundance	elevation	40	0.1575
		richness	elevation	42	0.0491
		mean DBH	elevation	42	0.2906
Uganda	National Park	abundance	mean DBH	618	-0.0911
		abundance	elevation	1	0.1387
		richness	abundance	618	0.7095
		richness	elevation	1	0.1667
		mean DBH	elevation	1	0.0258
Brazil	Bahia	abundance	richness	102	0.1961
		abundance	mean DBH	102	-0.7093
		abundance	elevation	102	0.0396
		richness	elevation	104	-0.4459
		mean DBH	elevation	104	0.3103
Chile	Northern Patagonia	abundance	richness	56	0.6906
		abundance	mean DBH	56	-0.0683
		abundance	elevation	49	-0.0308
		richness	elevation	49	-0.0794
		mean DBH	elevation	49	-0.0272
Myanmar	Wetphuyay	abundance	richness	41	0.7732
		abundance	mean DBH	41	-0.0936
		abundance	elevation	41	0.2669
		richness	elevation	43	-0.2135
		mean DBH	elevation	43	-0.1184
Peru	Río Abiseo National Park	abundance	mean DBH	27	-0.6209
		abundance	elevation	27	0.268
		richness	abundance	27	0.167
		richness	elevation	27	-0.8846
		mean DBH	elevation	28	-0.4196
Ecuador	Podocarpus National Park	abundance	mean DBH	27	-0.7831
		abundance	elevation	27	-0.0531
		richness	abundance	27	0.2649
		richness	elevation	27	-0.7469
		mean DBH	elevation	28	-0.3505

Country	Region	Response	Predictor	DF	Std. Estimates
Ecuador_dry	Western Ecuador	abundance	richness	44	0.5754
		abundance	mean DBH	44	-0.1691
		abundance	elevation	44	0.2697
		richness	elevation	46	0.5796
		mean DBH	elevation	46	-0.3288
Sweeden	Northern Sweeden	abundance	richness	97	0.6732
		abundance	mean DBH	97	0.1838
		abundance	elevation	97	0.0965
		richness	elevation	99	-0.0103
		mean DBH	elevation	99	0.083
Bolivia	Madidi National Park	abundance	mean DBH	37	-0.5519
		abundance	elevation	37	0.4762
		richness	abundance	37	0.4665
		richness	elevation	37	-0.2542
		mean DBH	elevation	38	-0.1045
Costa Rica	Costa Rica	abundance	mean DBH	90	-0.4856
		abundance	elevation	90	0.265
		richness	abundance	90	0.5426
		richness	elevation	90	-0.1009
		mean DBH	elevation	91	0.1217
Russia	Kola	abundance	richness	24	-0.0349
		abundance	mean DBH	24	-0.1093
		abundance	elevation	24	-0.1912
		richness	elevation	26	-0.221
		mean DBH	elevation	26	-0.0622
Bhutan	Toepisa	abundance	mean DBH	100	-0.3065
		abundance	elevation	100	0.1362
		richness	abundance	100	0.7282
		richness	elevation	100	-0.0828
		mean DBH	elevation	101	0.2024

Supplementary Material S2. Sensitivity analysis to the information criterion used for model selection.

Model selection in Structural Equation Models (SEM) can use different information criteria. Among the most popular, the Bayesian Information Criterion (BIC) has been supported over the traditional Akaike Information Criterion (AIC) in SEM statistics[16]. Other options based on the Fisher's C have been implemented as good candidates for model selection[17]. The author of the piecewiseSEM package in R[18], suggested the application of the C Information Criterion (CIC[19]) as a good alternative to the most classical AIC (though he termed CIC as AIC).

The piecewiseSEM package includes both indices BIC and CIC and so it is worth testing the sensitivity of our results to using one or the other criterion for model selection. To formally test this, we computed both BIC and CIC and analysed the relationship between them using a correlation test based on the Pearson moment correlation index. Supplementary Figure S1 shows that both indices yielded almost identical results.

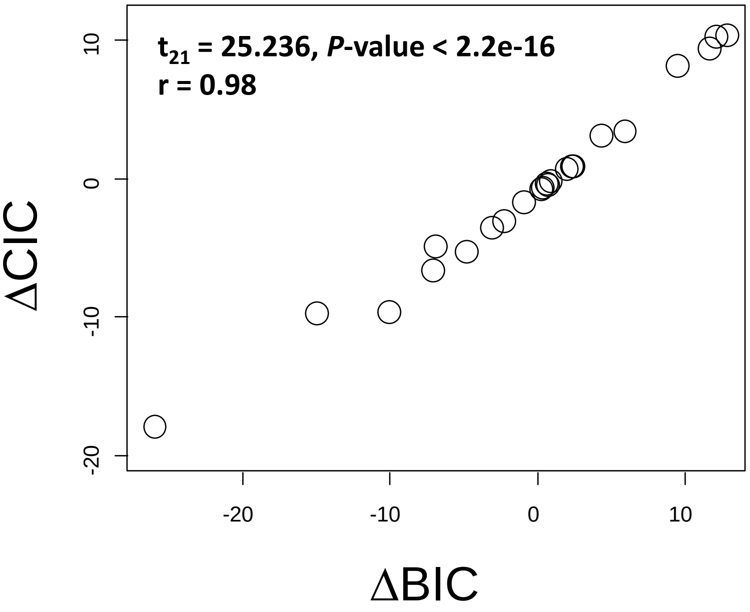

Supplementary Figure S1. Scatter plot for the relationship between the two potential candidate indices (CIC and BIC) to be applied for model selection in each region. Results of a correlation test based on the Pearson moment correlation coefficient (r) are also provided.

Supplementary Material S3. Sensitivity analysis to data harmonization.

Original data used in this research come from different sources: i.e. National Forest Inventories and previous research projects. This introduces uncertainties associated to the number of trees accounted for at the plot level if disparate protocols are applied to gather the different realities in the different regions. In particular, while some of the regional datasets consider trees with DBH higher than 5 cm, other set the threshold in 10 cm DBH. By analyzing each regional dataset independently, the issues related to this uncertainty should be largely alleviated. Yet, it is worth questioning whether these differences can affect the final outcomes of our research. To answer this question, we firstly harmonized the general dataset by including only trees with a DBH higher that 10 cm in each regional dataset. Then, we compared BIC (MSH vs MIH) according to the SEM model described above for every regional dataset in its original and harmonized versions separately.

To formally evaluate whether original or harmonized data introduce any discrepancy on the model outputs regarding the latitudinal patterns in ΔBIC, we fitted a linear model in which the ΔBIC is expressed as linear function of the interactive effects of latitude and the type of data (original/harmonized). This interaction implies that latitudinal patterns in ΔBIC varies depending on the type of data considered (i.e. the slope of the relationship change significantly between the original and harmonized data). Results using the Akaike Information Criterion corrected for small sample sizes (AICc[20]) rejected such interactive effects ($AICc_{interaction}$ = 306.9; $AICc_{no\text{-}interaction}$ = 305.5). Moreover, and even if influences of latitude on ΔBIC are assumed to remain constant, the ΔBIC could be comparatively higher or lower depending on the type of data (i.e. the main effects associated to the type of data influence the intercept in the linear model). To unveil this, we evaluated whether the type of data has a significant contribution to the ΔBIC

using a linear model. The results using the AICc showed negligible effects of the type of data on the ΔBIC ($AICc_{with\ type\ of\ data}$ = 305.5; $AICc_{without\ type\ of\ data}$ = 305.0).

As a second sensitivity analysis, we conducted a similar SEM model as the one used in the main text to evaluate the role of climate on the probability of prevalence of one hypothesis over the other (see methods in the main text), using the original and the harmonized data separately. The harmonized dataset yielded qualitatively and quantitatively very similar SEM results to the original dataset (see Figs. 4a and S3a). Moreover, observing the overlap of the 95% confidence intervals around the fitted marginal means on the relationship between ΔBIC and climatological NPP confirms that the original (green shaded area in Fig. S3b) and the harmonized (red shaded area in Supplementary Figure S3b) datasets provide very similar outcomes, supporting the robustness of our findings.

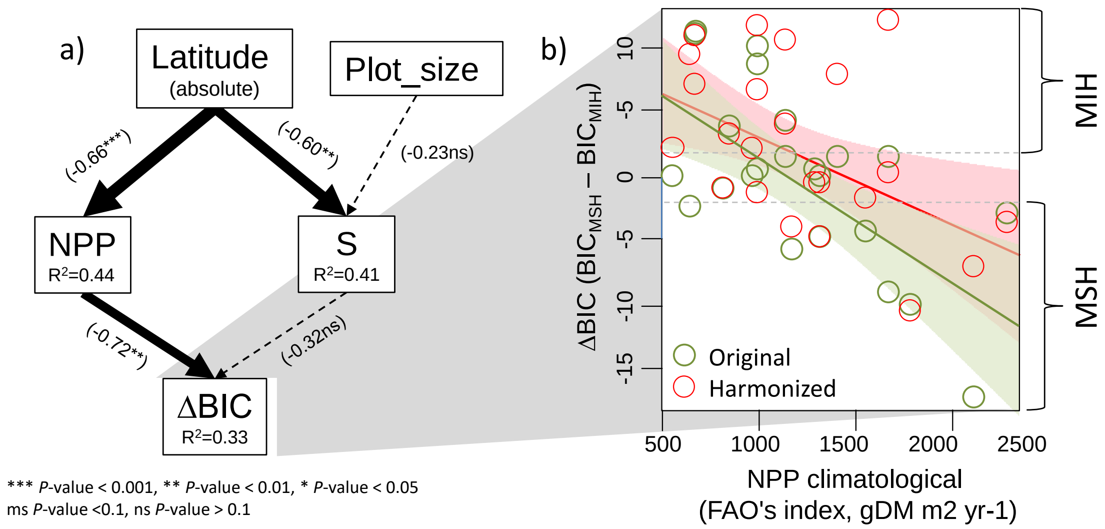

Supplementary Figure S2. Original and harmonized data support a similar pattern regarding the NPP- ΔBIC relationship. a) Graphical representation of the SEM outputs using harmonized data in which boxes represent the variables involved, arrows are illustrative of the causal paths, values in brackets denote the standardized coefficients (see legend of asterisks for P-value interpretation), and R^2 are the determination coefficients

for the different regression models considered in the SEM. Solid arrows are indicative of significant pathways whereas dashed ones imply no significant relationships (see also Fig. 4a). b) ΔBIC as linear function of NPP (FAO's climatological index). The shaded areas are illustrative of the 95% confidence interval around the marginal means (solid line in green) for original (green) and harmonized data (red). Dashed lines represent 2 units of ΔBIC above (+2) and below (-2) 0, which is indicative of equivalent support for both hypotheses (More Species (MSH) and More Individuals (MIH)). NPP: Net Primary Productivity; S: Species Richness; BIC: Bayesian Information Criterion.

Supplementary Material S4. Sensitivity analysis to environmental predictors other than elevation in the regional SEM.

We used elevation as a surrogate of environmental variability to account for gradients in species richness, mean tree size and abundance within regions. However, other environmental sources of variability might be considered to account for within-region patterns of species richness, mean tree size and abundance. To test whether accounting for other environmental sources of variability might distort the main pattern regarding the ΔBIC-NPP relationship, we conducted a sensitivity analysis using environmental variables other than elevation, namely NPP, Normalized Difference Vegetation Index (NDVI), soil Cation Exchange Capacity (CEC) and the combination of NPP and CEC. Moreover, we also included elevation in a second-order polynomial to account for potential hump-shaped gradients of species richness, mean tree size and abundance with elevation. We repeated the SEM analyses for every region considering each of the mentioned sources of environmental variability at a time. We did so to keep a feasible number of parameters to be estimated using the data available in each region. Finally, we studied the ΔBIC-NPP relationship for each set of environmental variables and the correlation in ΔBICs between among these sets.

As shown in Supplementary Figure S4 the pattern observed for the ΔBIC-NPP relationship maintains invariant. In every case, results point to a solid negative relationship between the ΔBIC and the climatological NPP which is in agreement with the reported pattern obtained using elevation in linear form. Moreover, the ΔBICs computed using the different environmental proxies were significantly and highly correlated (Pearson's r ranging from 0.78 to 0.96, P-value < 0.01 always, see Supplementary Table S3) regardless of the original or harmonized datasets (see also

Supplementary Material S3). Both results show that our results are largely insensitive to the environmental proxy used, confirming the robustness of the pattern detected.

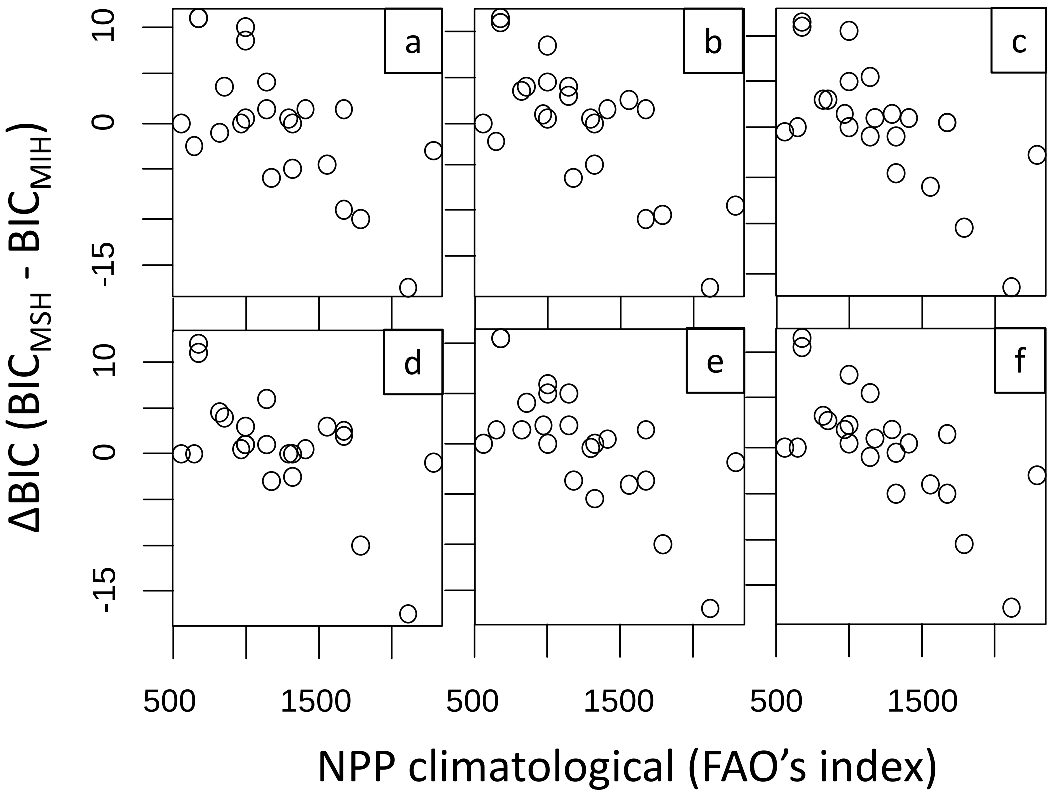

Supplementary Figure S3. Relationships between the NPP climatological and ΔBIC computed using different environmental predictor in the SEM models: a) elevation in linear form, b) elevation in second order polynomial, c) net primary productivity (NPP; 1km resolution grid), d) Normalized Difference Vegetation Index (NDVI) (1km resolution), e) Cation exchange capacity (CEC, 250 m resolution), and f) NPP and CEC together.

Supplementary Table S3. Correlation matrix (Pearson coefficient) for the ΔBIC obtained using the different environmental variables considered (see above) using the original data (a) and the harmonized data (b). All the correlations resulted highly significant at the *P-value* <0.01 level. Elev_linear: elevation in linear form; Elev_2nd poly: elevation in

second order polynomial; NPP: net primary productivity; NDVI: Normalized Difference Vegetation Index; CEC: Cation exchange capacity.

Elev_linear	Elev_2nd poly	NPP	NDVI	CEC	NPP+CEC	Original data (a)
1	0.93	0.91	0.81	0.96	0.92	Elev_linear
	1	0.83	0.85	0.91	0.90	Elev_2nd poly
		1	0.87	0.95	0.94	NPP
			1	0.91	0.90	NDVI
				1	0.96	CEC
					1	NPP+ CEC

Elev_linear	Elev_2nd poly	NPP	NDVI	CEC	NPP+CEC	Harmonized data (b)
1	0.93	0.90	0.84	0.92	0.93	Elev_linear
	1	0.85	0.78	0.89	0.85	Elev_2nd poly
		1	0.93	0.96	0.95	NPP
			1	0.86	0.93	NDVI
				1	0.94	CEC
					1	NPP+CEC

Supplementary Material S5. Sensitivity analysis to test for potential discrepancies in model outputs depending on which latitudinal proxy of productivity is considered (NPP climatological vs NDVI).

We used the NPP climatological (FAO'S index) as a good latitudinal composite of climatic influences on primary producers. This index is integrative of temperature and precipitation influences and has been applied in environmental science research previously (see for instance[21] in Nature). Nonetheless, it is worth considering other potential environmental proxies related to primary productivity. One such popular index is the NDVI (Normalized Difference Vegetation Index). This index has been recognized as a good surrogate for plant photosynthesis[22,23] and tree growth[24]. For this reason, we explored the relationships between NDVI, NPP and ΔBIC.

As shown in Supplementary Figure S5, NDVI and NPP are significantly and positively correlated. Both environmental proxies reflect a similar global pattern of productivity, one of them based on the reflected infrared radiation (NDVI) and the other one based on climatic influences, mostly temperature and precipitation. NPP seems to be a better predictor of ΔBIC than NDVI as shown by the Pearson correlation coefficient applied to both the original and the harmonized datasets. Importantly, the relationship between NDVI and ΔBIC is negative (significant or marginally significant depending on the type of data used) in support to the pattern reported in this manuscript. Moreover, NDVI is not a climatological index, but an environmental proxy integrative of climatological, but also other drivers of productivity at the global scale. This reinforce the idea that climate, over other environmental proxies at the latitudinal scale, is a major driver of the prevalence of Diversity effects over More Individuals towards the most productive forest biomes on Earth.

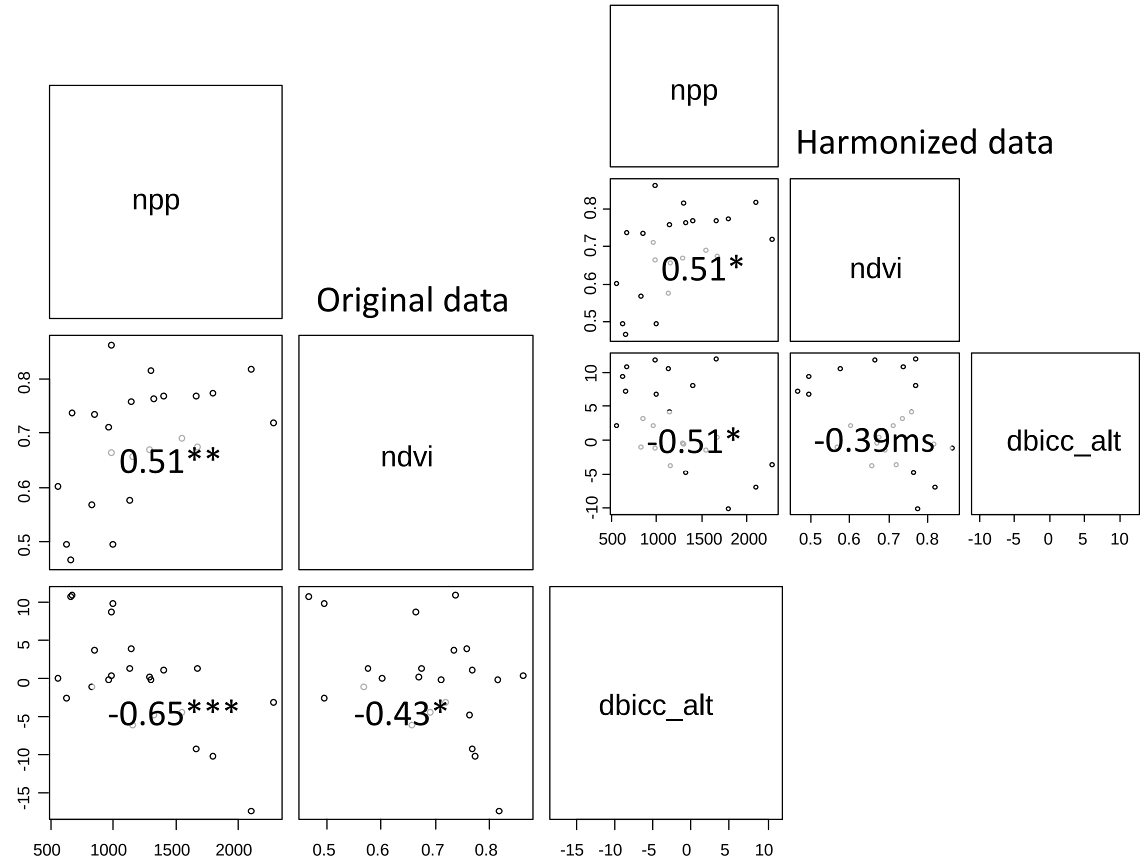

Supplementary Figure S4. Correlation matrices showing the relationship between ΔBIC, NDVI and NPP. Significance legend: ns P-value < 0.1, * P-value < 0.05, ** P-value < 0.01, ** P-value < 0.001. Correlograms were computed using the original data and the harmonized data separately. n = 23 forest regions.

Supplementary Material S6. Influence of the sampling size on the ΔBIC.

The number of plots varies from one dataset to another (see Supplementary Table S1) and this might artefactually influence ΔBIC estimations, and thus the interpretation of the pattern observed regarding the NPP-ΔBIC relationship. The correlation between the number of plots per region and the ΔBIC was, nonetheless, insignificant and very low (r = 0.09, P-valu*e*=0.68), which reject the influence of potential artifacts associated to the sample size in the different regions.

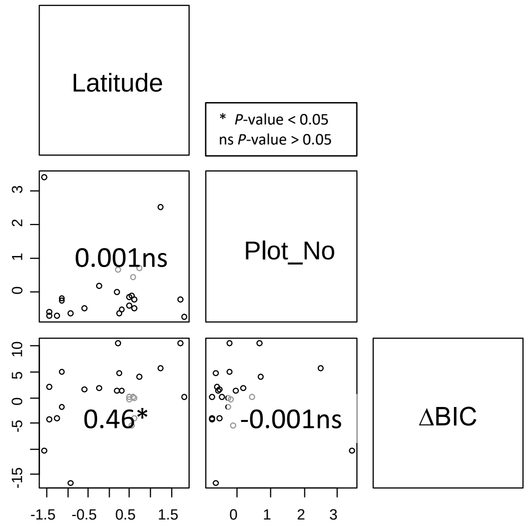

Supplementary Figure S5. Correlogram based on the Pearson moment correlation index for the relationships between Latitude, Plot_No (number of plots) and the ΔBIC. n = 23 forest regions.

Supplementary Material S7. Data selection and representativeness

Data selection criteria

We gathered forest plot data that meet the following criteria: 1) at least around 30 forest plots were sampled, to avoid overparametrization of subsequent models; 2) circular or rectangular sampling plots with identical sizes within each forest region (although variable among the different forest regions), to avoid area effects; 3) a clear spatial sampling design, to avoid the effect of spatial autocorrelation or treated it adequately; 4) a systematic sampling within plots to allow comparisons between them; 5) plots were distributed in extensive forested areas covering altitudinal gradients and; 6) plots were located in natural unmanaged forest to avoid anthropogenic impacts. To meet this last criterion, we only selected forest plots if they meet at least one of the following sub-criteria: 1) The natural unmanaged feature is clearly stablished in forest national inventories or; 2) plots were located within areas with a level of protection that preclude human use, including forest management or; 3) plots were located in areas of difficult access, far from densely populated human settlements and with evidence of low or inexistent industrial, farming and forestry activities. Following these criteria, we found 23 forest regions across the five forested continents on Earth. Of the 23 regions, five are located in North America (US), one in Central America (Costa Rica), six in South America (Brazil, Bolivia, Perú, Chile, and two in Ecuador), one in Africa (Uganda), one in Oceania (Australia), three in Asia (western Russia, Bhutan and Myanmar) and six in Europe (Sweden, Switzerland, 2 in France and 2 in Spain).

Description of each dataset

In this section we provided a brief description of the forested regions used. Forest datasets are ordered as in Supplementary Table S1.2.

1. We used 28 forest plots located in The Kola Peninsula (Rusia). Plots have 400 m^2 and were placed in the surroundings of the Murmanskin valtiollinen tundran rauhoitusalue Natural Park.
2. 132 plots located in the Sequoya National Park (USA) come from the USA national forest inventory (FIA) and have 168 m^2
3. We also used 229 plots of 168 m^2 coming from the USA national forest inventory (FIA), in this time located within Grand Canyon National Park
4. 101 plots of 1256 m^2 come from the Swedish national forest inventory and are located in the surroundings of the the Muddus/Muttos National Park (north Sweden)
5. We gathered 56 plots of 1963 m^2 from the Spanish national forest inventory. Plots were located within Sierra Nevada National Park.
6. We obtained 234 plots from the Swiss national forest inventory. Plots have 500 m^2 and are classified as not being perturbed at least in the last 100 years.
7. 160 plots of 500 m^2 were collected from Buthan national forest inventory. Plots are distributed in the surroundings of the Jigme Dorji National Park.
8. We used 491 plots located within Tongass National Forest (Alaska, USA). Plots have 168 m^2 and come from the USA national forest inventory (FIA).
9. We also used 197 plots from the USA national forest inventory (FIA). In this case, plots were located in contiguous protected areas (High Peaks Wilderness, Sargent Pond Wild Forest, West Canada Lake Wilderness and McKenzie Mountain Wilderness) located in the north of the state of New York.
10. We used 106 squared plots of 400m^2 randomly distributed in the surroundings of the Boqueirão da Onça National Park (Bahía, Brazil).

11. We gathered 48 plots of 400 m^2 located in the south-western of Ecuador nearby the border with Peru. This area has been heavily militarized due to recent border conflicts, which has allowed an almost pristine status of the forests.
12. 44 plots of 900 m^2 were retrieved from the Australian national forest inventory. We selected those plots that has not experienced a fire in at least the last 150 years and that were placed within Parks/Reserves or State Forests.
13. 61 plots of 706 m^2 and located in the Mercantour Nationa Park (France) were obtained from the France national forest inventory.
14. We retrieved 109 plots of 500 m^2 from the Chilean national forest inventory. Plots were classified as autochthonous mature forest (bosque autóctono maduro) and were located within a series of proximate national parks (del Alerce Andino, Corcovado, Vicente Rosales, Hornopirén) and a natural reserve (Parque Pumalín).
15. 98 plot of 706 m^2 and located in the Cévennes National Park (France) were retrieved from the French national forest inventory.
16. We gathered 117 plots from the Spanish national forest inventory. Plots have 1963 m^2 and were located within the Fuentes Carrionas Natural Park.
17. 75 Plots of 168 m^2 and located in the Klamath National Forest (USA) were obtained from the USA forest inventory.
18. We used 30 plots of 1000 m^2 located in the Podocarpus National Park (Ecuador).
19. Another 30 plots of of 1000 m^2 were sampled in the Río Abiseo National Park (Peru).
20. 62 plots were gathered from the national forest inventory of Myanmar. The plots have 500 m^2 and were located in the Wetpyuye Reserved Forest.
21. We retrieved data of 662 plots of 100 m^2 localted in the Budongo Forest Reserve (Uganda).

22. We used 44 plots of 1000 m^2 located in the Madidi National Park (Bolivia)
23. 96 plots of 1000 m^2 were collected from the Costa Rica national forest inventory. Plots classified as autochthonous mature forest and secondary forest were used.

Data representativeness

Following our search criteria, we gathered a total of 23 datasets distributed around the world. Using this sample of forests, we detected a clear pattern where the diversity effects hypothesis is supported toward more productive regions. Yet, it is worth questioning whether the sample of forest used is enough to robustly estimate the relationship between productivity and the support of the diversity effects hypothesis. To evaluated this, we conducted a bootstrap analysis over the linear regression of ΔBIC supporting the diversity effect hypothesis as a function of NPP. Bootstrap analysis is intended to estimate population parameters (in our case all world forests matching our search criteria) based on a given sample (i.e. the forests for which we found plot data)[25]. We resampled with replacement the forest datasets 10,000 times. In each bootstrap replicate, we computed a linear model of ΔBIC as a function of NPP, focusing on the statistical significance of the model, the coefficient of determination and the standardized regression coefficient. Bootstrap results strongly support the negative relationship between NPP and the diversity effect hypothesis. A total of 99.95% of bootstrapped replicates showed a significant negative relationship between NPP and ΔBIC (Supp. Fig. S6a). Moreover, we found a median $R^2 = 0.43$ (5th and 95th percentiles = 0.17 and 0.69; respectively, Supp. Fig. S6b) and a median standardized regression coefficient= -4.32 (5th and 95th percentiles = -6.71 and -2.09; respectively Supp. Fig. S6c). Therefore, these results show that the used sample of

forests provided robust results, confirming that the diversity forest hypothesis is supported toward the most productive regions on Earth.

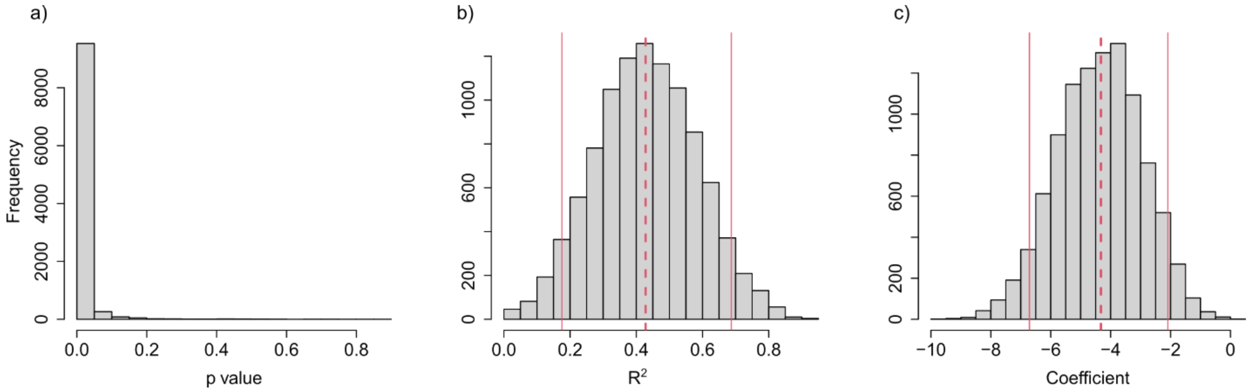

Supplementary Figure S6. Bootstrap results on the linear model of ΔBIC and NPP and for a) the significance of the model (P-value), b) the coefficient of determination (R^2) and c) the regression coefficient (Coefficient). The continuous red lines indicate the 5th and 95th quantiles whereas the dotted red line depicts the median of bootstrap replicates.

References

1. The Forest Inventory and Analysis (FIA) program of the U.S. Forest Service (https://www.fia.fs.fed.us/)

2. Fridman J., Holm S., Nilsson M., Nilsson P., Ringvall A. H., Ståhl G., 2014. Adapting National Forest Inventories to changing requirements – the case of the Swedish National Forest Inventory at the turn of the 20th century. Silva Fennica 48 no. 3 article id 1095. http://dx.doi. org/10.14214/sf.1095.

3. Alberdi, I., Sandoval, V., Condes, S., Cañellas, I., & Vallejo, R. (2016). El Inventario Forestal Nacional español, una herramienta para el conocimiento, la gestión y la conservación de los ecosistemas forestales arbolados. Revista Ecosistemas 25: 88-97.

4. Fischer C, Traub B .2019. Swiss National Forest Inventory Methods and Models of the Fourth Assessment. Springer Nature Switzerland.DOI: 10.1007/978-3-030-19293-8.

5. Dorji Y., Dorji R., Tshering P. 2016. National Forest Inventory Report: stocktaking Nation's Forest Resourceshttp://www.dofps.gov.bt/wp-content/uploads/2017/07/National-Forest-Inventory-Report-Vol1.pdf.

6. Joaquín Calatayud, Enrique Andivia, Adrián Escudero, Carlos J. Melián, Rubén Bernardo, Xavier Arnan, Nagore G. Medina, Luciano Pataro, Jorge A. Noriega,

Fernanda Alves, Cristina Aponte, Juan Ballesteros-Canovas, César Morales-Molino, Markus Stoffel, Martin Rosvall, Magnus Neuman, Arantzazu L. Luzuriaga, Isabel Draper, Rafael Molina-Venegas, Asier Herrero, Leandro Juen, Pablo Ferrandis, Alex Cea, Jaime Madrigal-González (2019). Positive associations among rare species drive their persistence in ecological assemblages. Nature Ecology and Evolution 4: 40-45.

7. Espinosa C.I., Cabrera O., Luzuriaga A.L., Escudero A. 2011. What factors affect diversity and species composition of endangered Tumbesian dry forests in southern Ecuador? Biotropica 43: 15-22.

8. Aponte C., Kasel S, Nitschke C, Tanase MA, Vickers H, Parker L, Fedrigo M, Kohout M, Ruiz-Benito P, Zavala MA, Bennett LT. 2020. Structural diversity underpins carbon storage in Australian temperate forests. Global Ecology and Biogeography 29: 789-802.

9. Robert, N., Vidal, C., Colin, A., Jean-Christophe, H., Hamza, N., Cluzeau, C. 2010. National forest inventories reports: France. In: Tomppo, E., Gschwantner, T.,Lawrence, M., McRoberts, R.E. (Eds.), National Forest Inventories—Pathways forCommon Reporting. Springer, pp. 207–221.

10. Sagardia, R. 2018. Inventario continuo de bosques nativos y actualización de plantaciones forestales. Ministerio de Agricultura, Gobierno de Chile (https://ifn.infor.cl/index.php/descargas-recursos/descargas/send/2-documentos-inventario-forestal/28-informe-recursos-forestal-en-chile-2018).

11. Bañares-de-Dios, G., Macía, M.J., Granzow-de la Cerda, Í., Arnelas, I., de Carvalho, G., Espinosa, C.I., Salinas, N., Swenson, N., Cayuela, L. 2020. Linking patterns and processes of tree community assembly across spatial scales in tropical montane forests. Ecology, (in press).

12. Myint M., Myint A.A., Min Thein, K., Su Mon M., Linn Aung H., Htoo K.K., Aung N., Oo L.M.M., Wai P., Aung P.P. 2018. Spatial lag distance of subplots at the terrestrial forests ecosystems for national level and subnational level forest inventory for UN-REDD national forest monitoring system of Myanmar. (Available upon request to maungmoe.myint@mnrii.com)

13. Plumptree, A.J. 1996. Changes following 60 years of selective timber harvesting in the Budongo Forest Reserve, Uganda. Forest Ecology and Management 89: 101-113.

14. Macía, M.J. 2008. Woody plants diversity, floristic composition and land use history in the Madidi National Parkn rain forests of Madidi National Park, Bolivia. Biodiversity and Conservation 17: 2671-2690.

15. Programa REDD/CCAD-GIZ - SINAC. 2015. Inventario Nacional Forestal de Costa Rica 2014-2015. Resultados y Caracterización de los Recursos Forestales. Preparado por: Emanuelli, P., Milla, F., Duarte, E., Emanuelli, J., Jiménez, A. y Chavarría, M.I. Programa Reducción de Emisiones por Deforestación y Degradación Forestal en Centroamérica y la República Dominicana (REDD/CCAD/GIZ) y Sistema Nacional de Áreas de Conservación (SINAC) Costa Rica. San José, Costa Rica. 380 p.

16. Lin, L. C., Huang, P. H., & Weng, L. J. (2017). Selecting path models in SEM: A comparison of model selection criteria. Structural Equation Modeling: A Multidisciplinary Journal, *24*(6), 855-869.

17. Shipley, B. (2013). The AIC model selection method applied to path analytic models compared using ad-separation test. Ecology, 94(3), 560-564.

18. Lefcheck, J. S. (2016). piecewiseSEM: Piecewise structural equation modelling in r for ecology, evolution, and systematics. Methods in Ecology and Evolution, 7(5), 573-579.

19. Cardon, M., Loot, G., Grenouillet, G., Blanchet, S. (2011). Host characteristics and environmental factors differentially drive the burden and pathogenicity of an ectoparasite: a multilevel causal analysis. Journal of Animal Ecology, 80(3), 657-667.

20. Hurvich, C. M., Tsai, C. L. (1989). Regression and time series model selection in small samples. Biometrika, 76(2), 297-307.

21. Lindgren, A., Hugelius, G., Kuhry, P. (2018). Extensive loss of past permafrost carbon but a net accumulation into present-day soils. Nature, 560(7717), 219-222.

22. Myneni RB, Keeling CD, Tucker CJ, Asrar G, Nemani RR (1997) Increased plant growth in the northern high latitudes from 1981 to 1991. Nature, 386, 698–702.

23. Nemani RR, Keeling CD, Hashimoto H et al. (2003) Climate-driven increases in global terrestrial net primary production from 1982 to 1999. Science, 300, 1560–1563.

24. Kaufmann RK, D'Arrigo RD, Laskowski C, Myneni RB, Zhou L, Davi NK (2004) The effect of growing season and summer greenness on northern forests. Geophysical Research Letters, 31, L09205.

25. Efron, B., & Tibshirani, R. J. (1994). An introduction to the bootstrap. CRC press.
