# Supplementary material for: Climate reverses directionality in the richness–abundance relationship across the World’s main forest biomes
Source: Nat Commun. 2020 Nov 6;11:5635. doi: 10.1038/s41467-020-19460-y (PMC7648646; doi:10.1038/s41467-020-19460-y)
Supplement: Supplementary file 3 — Reporting Summary [file 41467_2020_19460_MOESM3_ESM.pdf]

nature research

Corresponding author(s): JMG, NCOMMS-20-03110B

Last updated by author(s): Sep 24, 2020

Reporting Summary

Nature Research wishes to improve the reproducibility of the work that we publish. This form provides structure for consistency and transparency in reporting. For further information on Nature Research policies, see our Editorial Policies and the Editorial Policy Checklist.

Statistics

For all statistical analyses, confirm that the following items are present in the figure legend, table legend, main text, or Methods section.

n/a	Confirmed	
☐	☒	The exact sample size (n) for each experimental group/condition, given as a discrete number and unit of measurement
☐	☒	A statement on whether measurements were taken from distinct samples or whether the same sample was measured repeatedly
☐	☒	The statistical test(s) used AND whether they are one- or two-sided<br>*Only common tests should be described solely by name; describe more complex techniques in the Methods section.*
☐	☒	A description of all covariates tested
☐	☒	A description of any assumptions or corrections, such as tests of normality and adjustment for multiple comparisons
☐	☒	A full description of the statistical parameters including central tendency (e.g. means) or other basic estimates (e.g. regression coefficient) AND variation (e.g. standard deviation) or associated estimates of uncertainty (e.g. confidence intervals)
☐	☒	For null hypothesis testing, the test statistic (e.g. F, t, r) with confidence intervals, effect sizes, degrees of freedom and P value noted<br>*Give P values as exact values whenever suitable.*
☒	☐	For Bayesian analysis, information on the choice of priors and Markov chain Monte Carlo settings
☐	☒	For hierarchical and complex designs, identification of the appropriate level for tests and full reporting of outcomes
☐	☒	Estimates of effect sizes (e.g. Cohen's d, Pearson's r), indicating how they were calculated

Our web collection on statistics for biologists contains articles on many of the points above.

Software and code

Policy information about availability of computer code

Data collection	no software was used
Data analysis	The R environment version 3.6.1

For manuscripts utilizing custom algorithms or software that are central to the research but not yet described in published literature, software must be made available to editors and reviewers. We strongly encourage code deposition in a community repository (e.g. GitHub). See the Nature Research guidelines for submitting code & software for further information.

Data

Policy information about availability of data

All manuscripts must include a data availability statement. This statement should provide the following information, where applicable:

- Accession codes, unique identifiers, or web links for publicly available datasets
- A list of figures that have associated raw data
- A description of any restrictions on data availability

https://figshare.com/account/home (DOI 10.6084/m9.figshare.13072211)

Field-specific reporting

Ecological, evolutionary & environmental sciences study design

All studies must disclose on these points even when the disclosure is negative.

Study description	We considered a total of 23 forest regions across the five forested continents on Earth. Of the 23 regions, five are located in North America (US), one in Central America (Costa Rica), six in South America (Ecuador (2), Brazil, Bolivia, Perú, and Chile), one in Africa (Uganda), one in Oceania (Australia), three in Asia (eastern Russia, Bhutan and Myanmar) and six in Europe (Sweden, Switzerland, France (2) and Spain (2)). From each forest region, we used forest inventories from the corresponding National Forest Inventories (US, Costa Rica, Chile, Spain, France, Switzerland, Sweden, Bhutan and Myanmar) or forest inventories obtained for clearly-defined research purposes (Ecuador, Bolivia, Perú, Brazil, Uganda and Russia) with comparable protocols and clear sampling design. All together, these 23 forest regions comprise a total of 3,170 forest plots in which more than 84,000 individual trees have been sampled.
Research sample	For all the regions, we set a minimum number of 30 plots to ensure a sufficient number of replicates in the later statistical analyses. As mentioned above, we considered plots in natural forest only, out of selvicultural practices and human management. For this reason we encouraged data owners to select plots in natural reserves and national parks.<br>1. We used 28 forest plots located in The Kola Peninsula (Rusia). Plots have 400 m2 and were placed in the surroundings of the Murmanskin valtiollinen tundran rauhoitusalue Natural Park.<br>2. 132 plots located in the Sequoya National Park (USA) come from the USA national forest inventory (FIA) and have 168 m2<br>3. We also used 229 plots of 168 m2 coming from the USA national forest inventory (FIA), in this time located within Grand Canyon National Park<br>4. 101 plots of 1256 m2 come from the Swedish national forest inventory and are located in the surroundings of the the Muddus/Muttos National Park (north Sweden)<br>5. We gathered 56 plots of 1963 m2 from the Spanish national forest inventory. Plots were located within Sierra Nevada National Park.<br>6. We obtained 234 plots from the Swiss national forest inventory. Plots have 500 m2 and are classified as not being perturbed at least in the last 100 years.<br>7. 160 plots of 500 m2 were collected from Buthan national forest inventory. Plots are distributed in the surroundings of the Jigme Dorji National Park.<br>8. We used 491 plots located within Tongass National Forest (Alaska, USA). Plots have 168 m2 and come from the USA national forest inventory (FIA).<br>9. We also used 197 plots from the USA national forest inventory (FIA). In this case, plots were located in contiguous protected areas (High Peaks Wilderness, Sargent Pond Wild Forest, West Canada Lake Wilderness and McKenzie Mountain Wilderness) located in the north of the state of New York.<br>10. We used 106 squared plots of 400m2 randomly distributed in the surroundings of the Boqueirão da Onça National Park (Bahía, Brazil).<br>11. We gathered 48 plots of 400 m2 located in the south-western of Ecuador nearby the border with Peru. This area has been heavily militarized due to recent border conflicts, which has allowed an almost pristine status of the forests.<br>12. 44 plots of 900 m2 were retrieved from the Australian national forest inventory. We selected those plots that has not experienced a fire in at least the last 150 years and that were placed within "Parks/Reserves" or "State Forests".<br>13. 61 plots of 706 m2 and located in the Mercantour Nationa Park (France) were obtained from the France national forest inventory.<br>14. We retrieved 109 plots of 500 m2 from the Chilean national forest inventory. Plots were classified as autochthonous mature forest (bosque autoctono maduro) and were located within a series of proximate national parks (del Alerce Andino, Corcovado, Vicente Rosales, Hornopirén) and a natural reserve (Parque Pumalín).<br>15. 98 plot of 706 m2 and located in the Cévennes National Park (France) were retrieved from the French national forest inventory.<br>16. We gathered 117 plots from the Spanish national forest inventory. Plots have 1963 m2 and were located within the Fuentes Carrionas Natural Park.<br>17. 75 Plots of 168 m2 and located in the Klamath National Forest (USA) were obtained from the USA forest inventory.<br>18. We used 30 plots of 1000 m2 located in the Podocarpus National Park (Ecuador).<br>19. Another 30 plots of of 1000 m2 were sampled in the Río Abiseo National Park (Peru).<br>20. 62 plots were gathered from the national forest inventory of Myanmar. The plots have 500 m2 and were located in the Wetpyuye Reserved Forest.<br>21. We retrieved data of 662 plots of 100 m2 localted in the Budongo Forest Reserve (Uganda).<br>22. We used 44 plots of 1000 m2 located in the Madidi National Park (Bolivia)<br>23. 96 plots of 1000 m2 were collected from the Costa Rica national forest inventory. Plots classified as autochthonous mature forest and secondary forest were used.
Sampling strategy	Sample size for each forest region was set in, at least, 30 sampling plots, as two ensure enough sampling size for a three-predictor regression analysis
Data collection	The corresponding author got in contact with responsible persons in National Forest Inventories and researchers in the area of forest ecology
Timing and spatial scale	The different forest datasets were sampled in different time periods over the decades 1980-1990-2000. The spatial scale at each forest region can be identified with a landscape scale in which local altitudinal gradients are considered
Data exclusions	We gathered forest plot data that meet the following criteria: 1) at least around 30 forest plots were sampled, to avoid overparametrization of subsequent models; 2) circular or rectangular sampling plots with identical sizes within each forest region (although variable among the different forest regions), to avoid area effects; 3) a clear spatial sampling design, to avoid the effect of

	spatial autocorrelation or treated it adequately; 4) a systematic sampling within plots to allow comparisons between them; 5) plots were distributed in extensive forested areas covering altitudinal gradients and; 6) plots were located in natural unmanaged forest to avoid anthropogenic impacts. To meet this last criterion, we only selected forest plots if they meet at least one of the following sub-criteria: 1) The natural unmanaged feature is clearly stablished in forest national inventories or; 2) plots were located within areas with a level of protection that preclude human use, including forest management or; 3) plots were located in areas of difficult access, far from densely populated human settlements and with evidence of low or inexistent industrial, farming and forestry activities. These requirements were communicated to data owners for plot selection. We did not discarded plots afterwards.
Reproducibility	n/a
Randomization	This does not apply to our study sine data clustering represent spatial clustering according to the protocols of data sampling in the different forest inventories. For this reason, randomization does not apply here.
Blinding	n/a

Did the study involve field work? ☐ Yes ☒ No

Reporting for specific materials, systems and methods

We require information from authors about some types of materials, experimental systems and methods used in many studies. Here, indicate whether each material, system or method listed is relevant to your study. If you are not sure if a list item applies to your research, read the appropriate section before selecting a response.

Materials & experimental systems

n/a	Involved in the study	
☒	☐	Antibodies
☒	☐	Eukaryotic cell lines
☒	☐	Palaeontology and archaeology
☒	☐	Animals and other organisms
☒	☐	Human research participants
☒	☐	Clinical data
☒	☐	Dual use research of concern

Methods

n/a	Involved in the study	
☒	☐	ChIP-seq
☒	☐	Flow cytometry
☒	☐	MRI-based neuroimaging
